# Linking cortex and contraction—Integrating models along the corticomuscular pathway

**DOI:** 10.3389/fphys.2023.1095260

**Published:** 2023-05-10

**Authors:** Lysea Haggie, Laura Schmid, Oliver Röhrle, Thor Besier, Angus McMorland, Harnoor Saini

**Affiliations:** ^1^ Auckland Bioengineering Institute, University of Auckland, Auckland, New Zealand; ^2^ Institute for Modelling and Simulation of Biomechanical Systems, University of Stuttgart, Stuttgart, Germany; ^3^ Stuttgart Center for Simulation Sciences (SC SimTech), University of Stuttgart, Stuttgart, Germany; ^4^ Department of Exercise Sciences, University of Auckland, Auckland, New Zealand

**Keywords:** neuromuscular, corticospinal, proprioception, biophysical modelling, motor control, corticomuscular

## Abstract

Computational models of the neuromusculoskeletal system provide a deterministic approach to investigate input-output relationships in the human motor system. Neuromusculoskeletal models are typically used to estimate muscle activations and forces that are consistent with observed motion under healthy and pathological conditions. However, many movement pathologies originate in the brain, including stroke, cerebral palsy, and Parkinson’s disease, while most neuromusculoskeletal models deal exclusively with the peripheral nervous system and do not incorporate models of the motor cortex, cerebellum, or spinal cord. An integrated understanding of motor control is necessary to reveal underlying neural-input and motor-output relationships. To facilitate the development of integrated corticomuscular motor pathway models, we provide an overview of the neuromusculoskeletal modelling landscape with a focus on integrating computational models of the motor cortex, spinal cord circuitry, *α*-motoneurons  and skeletal muscle in regard to their role in generating voluntary muscle contraction. Further, we highlight the challenges and opportunities associated with an integrated corticomuscular pathway model, such as challenges in defining neuron connectivities, modelling standardisation, and opportunities in applying models to study emergent behaviour. Integrated corticomuscular pathway models have applications in brain-machine-interaction, education, and our understanding of neurological disease.

## 1 Introduction

From everyday tasks to highly-skilled athletic performance, movement is the result of a complex interaction between the central and peripheral nervous systems and muscle-tendon actuators. Neural circuits recruit skeletal muscle in a coordinated manner to produce movement and are connected through various feedback loops in the brain and spinal cord. Control of voluntary movement involves the interaction of multiple structures including the motor cortex, spinal cord circuits, skeletal muscles, and sensory organs. Signals from the motor cortex are carried via neurons of the corticospinal tract, and are integrated within spinal cord circuits, which in turn interact with skeletal muscles via *α*-motoneurons. Corticomotoneuronal cells (CM) of the motor cortex also make direct connections with *α*-motoneurons, particularly in the distal muscles of the upper limb (Lemon, 2008; Rathelot and Strick, 2009; Yeo et al., 2013). Sensory organs within the muscle and tendon provide the nervous system with information on the current state of the muscles, including their force, length, and contraction velocity, which modulates the descending drive from the cortex.

A mechanistic understanding of motor pathways, originating from the motor cortex, can lead to more effective diagnoses and personalised interventions to treat neuromuscular disorders. Neuromuscular pathologies may affect any and all parts of the motor pathway, impairing movement control and reducing quality of life. Degenerative disorders originating from supraspinal circuits, for example, impact the descending drive and can lead to ataxia (lack of coordination), dystonia (involuntary muscle spasms), and spasticity (stiffness, tightness of muscles) (Mukherjee and Chakravarty, 2010; Ganguly et al., 2021). Given the highly integrated and intricate nature of the motor pathway, the effects of neuromuscular disorders often spread beyond the afflicted region. For example, disorders such as amyotrophic and primary lateral scleroses affect “lower” and “upper” motoneurons, leading to the degeneration of muscle and muscle weakness, respectively (Tawil and Venance, 2011; Larsson et al., 2018). More generally, motoneuron atrophy also alters a muscle’s fibre composition during ageing (sarcopenia) (Larsson et al., 2018). Many other diseases also originate from the brain and have a downstream effect on muscle contraction including cerebral palsy, stroke, Parkinson’s, and Huntington’s disease. These are a few illustrative examples of movement disorders involving the motor pathway.

Experimental techniques capture essential data to investigate the properties and function of various components in healthy and diseased states, which provides valuable insight to understand the motor pathway. However, given the complexity and interdependency of the motor pathway, combined with technical and ethical limitations, experiments on isolated parts of the pathway leave many unanswered questions regarding the input/output relationships of the system. For example, transcranial magnetic stimulation (TMS) has been successfully used as a measure of cortical excitability to assess patients with stroke and epilepsy, but its limited ability to only impact the cortical level and the large variability in responses is a major limitation in exploring the role of subcortical components in the motor pathway (Blicher et al., 2009; Badawy et al., 2014). Neuromuscular activity and architecture can be characterised by various means such as: electrophysiological techniques for muscle activity, motor unit number, and spike train estimates, medical imaging for motor unit anatomy, intramuscular pressure for muscle activity, and joint force estimation for determining motor unit twitch properties (Troiani et al., 1999; Holobar et al., 2010; Csapo et al., 2015; Ateş et al., 2018; Lapatki et al., 2019; Rohlén et al., 2020). Each experimental technique is accompanied by certain drawbacks; for example, electrophysiological techniques only capture a fraction of active motor units, are prone to cross-talk, and are sensitive to movement artefacts (Negro et al., 2016a; Yavuz et al., 2018; Lapatki et al., 2019; Mesin, 2020), and medical imaging techniques are typically constrained to low contraction levels or non-functional poses (Csapo et al., 2015; Rohlén et al., 2020). These limitations highlight a need for a computational approach, which incorporates experimental data to understand the motor pathway.

Mathematical and computational models provide an alternative means to investigate the motor pathway. The process of describing physiological systems as mathematical models requires systematic and detailed analyses of the system and the identification of relevant inputs, outputs and “physiological parameters”. The advantage of mathematical models is the ability to perform *in silico* experiments to test hypotheses, which is especially beneficial to intricately connected systems such as the motor pathway. However, integrating the various components of the motor pathway remains a challenge, and most approaches only consider a limited number of parts or combinations of the physiological components involved in movement generation. A common example is to include *α*-motoneuron pools in skeletal muscle models to predict force generation as a function of *α*-motoneuron firing times or neural input (e.g., Röhrle et al., 2008; Farina et al., 2017; Sartori et al., 2017; Volk et al., 2021). Integrating afferent feedback from proprioceptive sensory organs within spinal cord neuronal circuits is another example of combining different components of the motor pathway to understand inherent behaviour, such as the relationship between spinal circuit connectivity and postural control (e.g., Stienen et al., 2007; Raphael et al., 2010; Elias et al., 2014; Dideriksen et al., 2015; Sreenivasa et al., 2015; Moraud et al., 2016; Aoyama and Kohno, 2022; Kapardi et al., 2022). By beginning to incorporate more physiologically realistic circuits further upstream to muscle, neuromusculoskeletal models can become more useful in answering questions about motor pathologies and motor control.

Our aim is to facilitate the development of a generalised, integrated model of the motor pathway from motor cortex to muscle for the voluntary control of movement, i.e., the corticomuscular pathway, by building on existing models. Holistic corticomuscular pathway models, which aim to represent the interconnections within the pathway rather than isolated parts, could provide distinct insights. For example, investigating the contribution of the cortex to long-latency responses of reflexes could help to distinguish spinal and cortical contributions to reflexes (Matthews, 1991; Reschechtko and Pruszynski, 2020). Elucidating relationships between motor cortex excitability and motor output can provide a more mechanistic understanding of underlying clinical presentations enabling design of more effective rehabilitation protocols (Derosiere et al., 2020). Clinically, modelling of neurosurgical procedures could help guide treatments, such as selective dorsal rhizotomy, which involves the cutting of dorsal roots to mitigate spasticity in patients with cerebral palsy by reducing sensory feedback (Enslin et al., 2019). Furthermore, the relationship between cortical activity and muscle activity, and ultimately the role of the motor cortex in the generation of movement can be investigated (Scott, 2008). This, certainly incomplete, list of research questions reveals the potential of a holistic approach to modelling the corticomuscular pathway.

Despite advances towards integrated modelling of the neuromuscular system, the contribution of the central nervous system (CNS), or any supraspinal input, is rarely considered. Recently, attempts have been made towards modelling more complex representations of the corticomuscular pathway (Teka et al., 2017; James et al., 2018; Pérez Fernández et al., 2021). These models are important milestones towards a holistic approach of motor pathway modelling but are limited in scope since they either consider very specialised pathways, such as the control of eye movement (James et al., 2018; Pérez Fernández et al., 2021), or use simplified model components (Teka et al., 2017). Thus, these modelling frameworks are not suited to investigate the complex interplays within the corticomuscular pathway and related pathologies, highlighting the need for an integrated model.

This work provides an overview of the modelling landscape of components that make up the pathway from cortex to muscle, namely, the motor cortex, spinal circuits, skeletal muscle, and proprioceptive sensory organs. Section 2 provides the physiological background for each component. Section 3 addresses the mathematical modelling of the corticomuscular pathway. Here, each subsection synthesises typical modelling approaches and provides exemplary model equations, together with relevant parameterisation and proposals for input-output parameters to adjacent component models. Section 4 concludes with a discussion on model implementation, validation, and future opportunities.

## 2 Physiological background of the corticomuscular pathway

**FIGURE 1:**
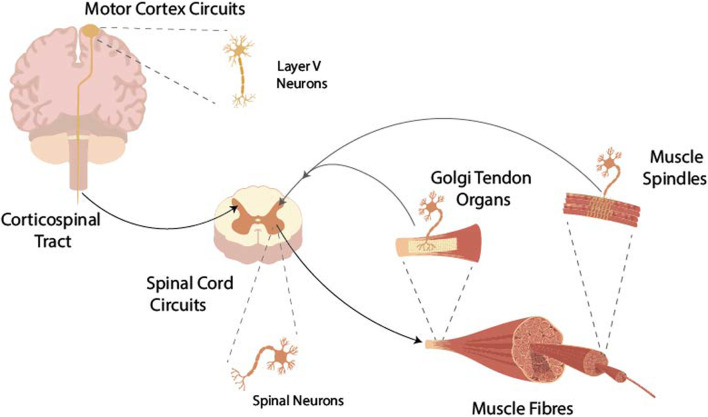
Intentional movement commands from the motor cortex are transferred via the axons of layer V pyramidal neurons, which make up the corticospinal tract, to the neurons of the spinal cord. From these, the *α*-motoneurons finally activate muscle fibres. Muscle contraction is monitored by Golgi tendon organs and muscle spindles and sensory information is fed back to the spinal circuits modulating the activity of the neurons in the spinal cord.

The motor cortex issues volitional movement commands as a result of intricate interactions involving internal network dynamics and connections with other brain areas (Sauerbrei et al., 2020; Logiaco et al., 2021). These movement commands, in the form of action potentials, travel down the spinal cord and synapse with a variety of spinal interneurons as well as *α*-motoneurons (Lemon, 2008; Baldissera et al., 2011). A number of descending pathways, such as the reticulospinal pathway, progress via brainstem nuclei, and while these are clearly important for motor control (Sheean, 2002; Li et al., 2019), they are beyond further scope of this text. Each *α*-motoneuron innervates a specific set of muscle fibres (forming the motor unit), thus controlling muscle contraction. The state of the musculoskeletal system is monitored by a number of sensory organs in muscle and tendon, which send signals back to the central nervous system where this afferent information is used to adapt the movement commands. This work focuses on the feedback pathways within the spinal cord circuits; for models of feedback in motor control involving supraspinal circuits see Scott (2016). Figure 1 shows the overview of the components in the motor pathway considered. In the following section, an overview of the relevant physiology of components making up the corticomuscular pathway is given. A more detailed anatomical and physiological description of the components of the neuromuscular system and movement control can be found in Kandel et al. (2021).

### 2.1 Motor cortex

The motor cortex is directly involved in the generation of muscle contraction. The experiments of Fritsch and Hitzig in the mid-19th century established that electrically stimulating the frontal regions of the cortex elicits movement, mainly in the contralateral side of the body (Gross, 2007; Hagner, 2012). By the 1930s, mapping of body representations in the motor and sensory areas was carried out through electrical stimulation applied to the exposed cortical surface of epileptic patients in surgery (Penfield and Boldrey, 1937). Presently, studies using non-invasive methods, including TMS, link the stimulation of neurons in the motor cortex to evoked responses in the peripheral muscle (Badawy et al., 2014; Volz et al., 2015). Neurons in the motor cortex make up a significant proportion of the corticospinal tract (CST), which is one of the primary motor pathways involved in voluntary movement of humans (Lemon, 2008). CST neurons then connect to spinal interneurons and *α*-motoneurons and then on to muscle, resulting in movement.

**FIGURE 2:**
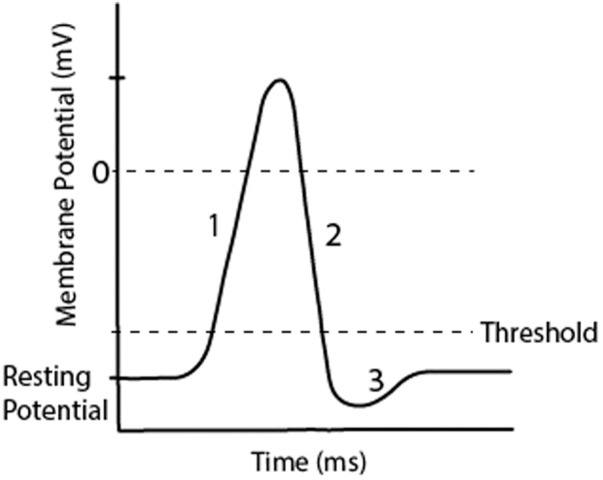
A representation of an action potential in a neuron, which occurs when the membrane reaches threshold from accumulated post-synaptic potentials. Regions of the graph show 1) Depolarisation, 2) Repolarisation, 3) Hyperpolarisation. This transient increase in membrane potential occurs over less than 3 ms.

Neurons of the central and peripheral nervous system send and receive information through electrochemical signalling. The membrane of a neuron acts to separate charges between the inside and outside of the cell. It also contains channels which control ion flow and pumps that maintain concentrations of ions inside and outside of the cell. At rest, there is typically a high concentration of extracellular Na^+^ and Cl^−^ and intracellularly a high concentration of K^+^. This difference in ion concentrations creates an electrical potential. When the electrical potential of the neuron reaches a certain threshold a sudden and transient depolarisation of the cell occurs, known as an action potential or ‘spike’. When a pre-synaptic neuron fires an action potential, it travels down the axon and crosses the synapse with post-synaptic neurons resulting in a post-synaptic potential, either increasing or decreasing the membrane potential of the post-synaptic neurons. The membrane potential ranges from approximately −90 mV when it is at rest, to approximately 50 mV during an action potential. The threshold is approximately −60 mV, though these values depend on species and cell type (Dayan and Abbott, 2001). A typical representation of an action potential is shown in Figure 2, note that the shape of an action potential can vary widely (Krutki et al., 2022).

In 1 mm^2^ of surface area in the motor cortex there are approximately 50,000–90,000 neurons (Young et al., 2013; Collins et al., 2016). Neurons in the cortex have a diverse range of morphologies and electrophysiological behaviour. Neurons can be categorised by their connectivity: those that are connected within the cortex are refered to as corticocortical neurons and those that make connections outside the cortex include corticothalamic, corticospinal or corticomotor neurons (Oswald et al., 2013). Neurons can also be distinguished by their effect on post-synaptic ion channels resulting from neurotransmitter release. Excitatory neurotransmitters such as glutamate increase a cell’s likelihood to fire an action potential, whereas inhibitory neurotransmitters decrease a cell’s likelihood to fire an action potential. Excitatory and inhibitory post-synaptic potentials are received and integrated mostly in the dendrites of a post-synaptic neuron which, if it reaches threshold, triggers an action potential to propagate along the post-synaptic neuron’s axon. Therefore, the connectivity of neurons can play a significant role in the propagation of action potentials and information in the neuronal circuits involving movement (Udvary et al., 2022). For more information on neuron behaviour and circuits, see Dayan and Abbott (2001).

Experiments involving microstimulation or tracer injections have uncovered a somatotopic organisation of the motor cortex with areas effecting the lower limb located more medially and the areas controlling the trunk, upper limb, face and hands extending distally [(He et al., 1995; Mitz and Wise, 1997; Park et al., 2001), but very recently revised by (Gordon et al., 2022)]. The face and hands, which have finer motor control, are represented by larger surface areas of the cortex (Schieber, 2001). Recent research has also showed distributed and overlapping representations of muscles and body parts in specific regions, for example, in the upper limb region, digit, wrist, elbow and shoulder areas show patchy (0.25–1.0 mm radius) connectivity patterns (Schieber, 2001; Rathelot and Strick, 2009; Hatsopoulos, 2010; Card and Gharbawie, 2020). This suggests that the intrinsic networks may be functionally connected according to end-effectors (i.e., muscles) within the somatotopic organisation of the motor cortex (Card and Gharbawie, 2020).

Evidence of the cortex having a layered structure dates back to histological staining carried out by Broadmann and Cajal who delineated the cortex into six layers differing in populations of cell bodies and cell types (Castro-Alamancos, 2013). In the motor cortex, layer V is the most prominent and contains the bodies of large pyramidal cells which serves as the main ‘output’ layer to other movement areas in the brainstem and spinal cord (Li N. et al., 2015) (for review of descending tracts see Lemon, 2008). Layer I mainly contains projections and no cell bodies. Thalamic input projections to the motor cortex occur across all layers, densely to layer III–V and less densely to layer VI (Tanaka, 2016). Experimental evidence has also suggested the idea of vertical columns in the cortex but the functional properties of this spatial structure have not been determined (Horton and Adams, 2005; Georgopoulos et al., 2007).

The motor cortex instigates voluntary muscle contraction of the body mainly thorough direct corticomotor (CM) connections to *α*-motoneurons and the corticospinal tract (CST) via spinal interneuron circuits (Lemon, 2008; Fregosi et al., 2019). The CST is the major anatomical pathway for transmitting movement related information from the brain to the spinal cord (Figure 1). Only approximately 0.05% of cells in the motor cortex contribute to the CST (Keller, 1993). However, projections from the motor cortex are the largest contributor to the CST, making up 30%–50% of the descending pathway, which contains approximately 1 million myelinated axons (Keller, 1993; Saliani et al., 2017). Other contributions to the CST come from secondary motor areas including the premotor and supplementary motor areas, as well as the somatosensory cortex (Dum and Strick, 2005; Lemon, 2008). Approximately 90% of neurons of the CST decussate in the brain stem, resulting in contralateral control (Lacroix et al., 2004; Natali et al., 2022). CST axons project to the grey matter of the ventral horn of the spinal cord forming synapses with interneurons and *α*-motoneurons along multiple levels of the spinal column and multiple *α*-motoneuron pools within levels.

### 2.2 Spinal circuitry

In the spinal circuits, signals from motor areas in the brain as well as from peripheral sensory organs are integrated and processed (Kandel et al., 2021). The *α*-motoneurons located in the ventral horn of the spinal cord, receive these signals and activate muscle contraction (Figure 1). Some afferent nerves, originating from peripheral sensory organs in muscles, form direct (i.e., monosynaptic) connections to the *α*-motoneurons. However, most afferent nerves terminate on interneurons which then connect, directly or indirectly via several other interneurons to *α*-motoneurons, creating polysynaptic pathways. These pathways excite or inhibit *α*-motoneurons, depending on the type of sensory organ and the muscle it is located in (Baldissera et al., 2011). Thereby, interneurons are themselves regulated by supraspinal inputs (Baldissera et al., 2011). In addition, signal transmission from central as well as peripheral pathways differs between flexor and extensor muscles (Yavuz et al., 2018; Castle-Kirszbaum and Goldschlager, 2021). Further, Renshaw cells deliver direct recurrent inhibition to *α*-motoneurons (Windhorst, 1990). The totality of these pathways determines the generation of action potentials along the motor axon to initiate contraction in the muscle fibres. Spinal circuits also potentially contribute to the recruitment of muscle synergies, i.e., groups of multiple muscles activated concurrently, and in central pattern generators, particularly in locomotion (Duysens and de Crommert, 1998; Tresch and Bizzi, 1999; Dietz, 2003).

**FIGURE 3:**
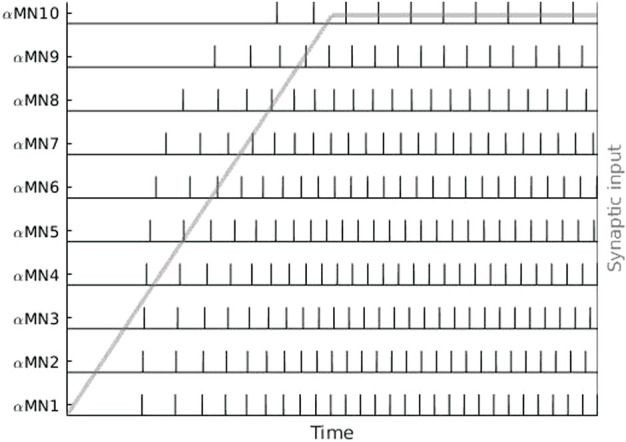
Sequence of action potentials of ten *α*-motoneurons (*α*MN) in response to a ramp-and-hold type of synaptic input (grey). Cell size and recruitment threshold increase exponentially from *α*MN1 to *α*MN10. The number of recruited *α*-motoneurons and their respective firing rates increase with increasing synaptic input, illustrating the size principle and onion skin firing scheme.

Each *α*-motoneuron innervates a specific set of muscle fibres, which is called a motor unit (MU) (Section 2.3). The number of innervated muscle fibres is proportional to the size of the neuron, resulting in different sized motor units (Heckman and Enoka, 2012). A pool of *α*-motoneurons, which consists of all the motoneurons that innervate a single muscle, typically contains a larger number of smaller neurons (Gustafsson and Pinter, 1984; Powers and Binder, 1985). The central nervous system uses two strategies to modulate the force that is produced by a specific muscle: recruitment increases the number of active MUs and rate coding increases the activity of a specific MU. With increasing excitatory synaptic input into a pool of *α*-motoneurons, motor units are usually recruited in an ordered manner, from smallest (low-threshold) to largest (high-threshold), following Henneman’s size principle (Henneman et al., 1965). At the same time, increasing synaptic input leads to an increase in the frequency of action potentials generated by a specific neuron. Small neurons, which are recruited earlier, usually fire action potentials at higher rates compared to large motoneurons. This is known as the onion skin principle (De Luca and Hostage, 2010). The size and onion skin principle are illustrated in Figure 3. Note that recent evidence suggests that Henneman’s size principle might be a simplification, and that more complex patterns of motor unit recruitment are possible (Marshall et al., 2022). Further, it is widely accepted that motoneurons innervating one and the same muscle share most of the inputs (Negro et al., 2016b; Del Vecchio et al., 2022). Interestingly, recent research suggests that this approach should be redefined to functional groups of motor units spanning more than one muscle (Hug et al., 2023).

More detailed information on motor units can be found in Heckman and Enoka (2012) and for an elaborate review of the neural circuits within the spinal cord see Baldissera et al. (2011).

### 2.3 Skeletal muscle

Skeletal muscle can rapidly contract and generate force in response to recruitment via the central and peripheral nervous systems (Figure 1). The tremendous range of movements that the human body is capable of is partly made possible by the variety of muscle shapes and architecture. For example, the soleus of the lower legs may be thousands of times larger than the lumbricals of the hand. Despite this variety, individual muscles and tendons (musculotendon complex) share commonalities that are adapted to each individual joint. Skeletal muscle is a hierarchical structure of repeating units—these are, from the largest (at the centimeter range) to the smallest (at the micrometer range): muscle fascicles, fibres, myofibrils, and sarcomeres. The contractile elements are surrounded by connective tissues: the epimysium, perimysium, and endomysium, which surround the muscle, fascicles, and fibres, respectively. Connective tissues are the primary means of force transmission, both laterally within the muscle and longitudinally towards the tendons (Purslow, 2010; Turrina et al., 2013).

Muscle fibres can be separated by histochemical staining intensities, which generally correspond to their rate of contraction and fatiguability. Commonly, three classifications are used, type-I, -IIA, and -IIB, but there may be up to seven, with each classification increasing in contractile speed and fatiguability (Scott et al., 2001). A single *α*-motoneuron typically innervates multiple muscle fibres. The neuron and its fibres are referred to as a motor unit. Muscles have varying numbers of motor units (equalling the number of *α*-motoneuron axons per muscle), e.g., ranging from five in the rectus lateralis to approximately 1750 in the gastrocnemius. Furthermore, motor units within a muscle vary in size according to the number of muscle fibres they contain, termed as a motor unit’s innervation ratio. The largest motor unit in a muscle may contain up to eighty times the fibres of the smallest one. The remaining motor units typically follow an exponential distribution between these extremes (Heckman and Enoka, 2012, and references therein). Furthermore, the contractile properties of motor units varies due to their fibre type composition. The three-fold classification of muscle fibres is thus commonly applied to motor units also, with type-I, IIA, and IIB fibres comprising type S, FR, and FF motor units, respectively. The variability in contraction dynamics and fatigability means that certain motor units are better suited for certain tasks, e.g., type S, FR, and FF motor units for posture maintenance, walking and running (Henning and Lomo, 1985).

The distribution of a motor unit’s fibres (or motor unit anatomy) is typically not uniform within the muscle but rather limited to a fraction of the muscle. Motor unit anatomy can be altered due to factors, such as age, pathological conditions, and level of physical (in) activity (e.g., Lexell and Downham, 1991; Messi et al., 2016). Despite this large degree of variation, motor unit anatomy can be generalised as locally confined to a region of the muscle, overlapping with multiple other territories, irregularly shaped, and as having varying degrees of fibre-type clustering (e.g., Bodine-Fowler et al., 1990; Lexell and Downham, 1991; van Dijk et al., 2016). Muscle force production is a complex interplay between various factors, including its geometry, material properties, fibre-arrangement, and motor unit recruitment and anatomy.

The neural system does not recruit individual muscle fibres but rather groups of fibres simultaneously, i.e., via recruitment of motor units. An action potential from the *α*-motoneuron arrives at the neuromuscular junctions of all fibres that it innervates. When recruited, excitation-contraction coupling initiates at the muscle fibre’s neuromuscular junction, leading to (local) depolarisation of the muscle fibre. Sarcomeres are composed of thin (myosin) and thick (actin) filaments, and is the site at which chemical energy is converted into mechanical force. This process is referred to as cross bridge cycling, and is initiated by changes in ion concentrations within the cell as a result of muscle (fibre) recruitment via the *α*-motoneurons. The amount of force produced within the sarcomere depends in part on the degree of overlap between the thick- and thin-filaments and the rate of contraction. Briefly, as sarcomeres lengthen or shorten beyond their optimal length, filament overlap decreases and fewer cross bridges can be formed, leading to lower force production (Ramsey and Street, 1940; Gordon et al., 1966). Furthermore, as the rate of contraction increases, myosin heads may no longer find actin attachment sites and a lower force is produced (Hill, 1938). The action potential propagates outwards towards the fibre’s distal ends. In the wake of the action potential, an increase in Ca^2+^ concentration occurs in the sarcoplasm, leading to cross bridge cycling in muscle fibres.

### 2.4 Proprioceptive feedback

The ability to perform coordinated movements is closely linked to the ability to sense position. The term proprioception is commonly used to describe the awareness of limb position and movement, force, effort and balance (Proske and Gandevia, 2012). Several sensory organs provide the central nervous system with proprioceptive information. In particular, muscle spindles and Golgi tendon organs (GTOs) (Figure 1) play an important role in motion control, since their activity is closely related to muscle activity.

Muscle spindles can be found in almost all skeletal muscles and make the biggest contribution to proprioception (Macefield and Knellwolf, 2018). They are sensitive to length changes of their parent muscle, i.e., muscle fibre stretch. The number of muscle spindles in human muscles can vary between less than ten and more than 1,000, depending on muscle size and function (Banks, 2006). Anatomically, muscle spindles are arranged in parallel to and embedded within the main, or extrafusal, muscle fibres. Three different types of intrafusal fibres experience length changes whenever their parent muscle changes in length. The different types of fibres have different viscoelastic properties that make them differently sensitive to muscle length and velocity. The sensory information is transferred to the neural circuits in the spinal cord via two types of afferent nerves, Ia (primary) and II (secondary) afferents. Thereby, Ia afferents are unique in that they form monosynaptic connections to *α*-motoneurons of the same (homonymous) muscle (Stauffer et al., 1976; Watt et al., 1976). Further, Ia and II afferents connect di- and polysynaptically to the homonymous as well as to other (heteronymous) muscles, i.e., synergists and antagonists (Scott and Mendell, 1976; Watt et al., 1976). In general, muscle spindles excite homonymous and synergistic muscles and inhibit antagonists (Scott and Mendell, 1976; Watt et al., 1976). In particular, the excitatory Ia input to motoneurons contributes to a considerable extent to muscle activation (Gandevia et al., 1990; Hiebert and Pearson, 1999). Muscle spindle activity is modulated by the fusimotor system, which comprises two types of spinal neurons, namely, static and dynamic *γ*-motoneurons (Matthews, 1962). By activating muscle spindles, *γ*-motoneurons modulate the spindles’ sensitivity and, importantly, ensure that spindles remain responsive during muscle contraction (Macefield and Knellwolf, 2018).

Golgi tendon organs are located at the musculotendinous interface and each GTO lies in series with a number of muscle fibres (Schoultz and Swett, 1972). GTOs are sensitive to the force produced by these muscle fibres (Anderson, 1974). Thereby, each GTO is sensitive to contractions of several motor units and each motor unit is monitored by several GTOs (Jami, 1992). Each tendon organ is usually innervated by a single afferent nerve fibre, a Ib afferent (Schoultz and Swett, 1972). Ib afferents form di- or trisynaptic inhibitory connections to the *α*-motoneuron pool of the homonymous muscle (Jami, 1992). The number of GTOs per muscle is in general smaller than the number of spindles, with approximately 0.7 GTOs per muscle spindle (Jami, 1992). For information beyond this short summary the reader is referred to Proske and Gandevia (2012) and Kandel et al. (2021).

## 3 Mathematical modelling of the corticomuscular pathway

**FIGURE 4:**
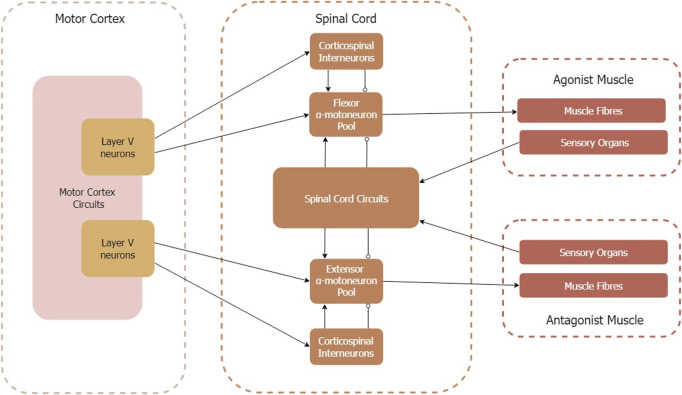
Circuit diagram of corticomuscular pathway model components. Arrows indicate excitatory connections, circles indicate inhibitory connections. Note that the spinal circuits transmit sensory organ signals differently to flexor and extensor *α*-motoneuron pools (for details see Section 2.4).

This work aims to describe how state-of-the-art models of the motor cortex, spinal circuits, skeletal muscle, and proprioceptive sensory organs can be integrated to form a model of the corticomuscular pathway. Figure 4 provides an overview of the model components and illustrates the relevant interfaces. We consider the voluntary and sensory control of an antagonistic muscle pair. Therefore, neither synergistic muscle activity nor rhythmic muscle activity (i.e., emerging from central pattern generators) is considered. Additionally, proprioceptive feedback is considered on the spinal cord level only, i.e., spinal-cortical pathways are beyond the scope of this work.

We define mathematical models as both stochastic and deterministic models as opposed to experimental or animal models. Modelling approaches to physiological systems can be classified broadly into two categories: phenomenological and biophysical. Phenomenological models aim to describe and relate input/output variables to match experimental data, irrespective of the underlying mechanism, i.e., a “black-box” approach. In contrast, biophysical models use relevant physiological knowledge to describe the underlying mechanism(s). Phenomenological models may be useful for model reduction, and may be the only viable choice when computational costs are a factor. On the other hand, mechanistic physiological representations may be paramount to understand emergent behaviour in integrated, complex systems.

This section is structured as follows; first motor cortex modelling is covered, including isolated neuron models, approaches to form neural networks, and the corticospinal pathway (Section 3.1). Second, spinal circuitry models are described, with a focus on spinal neurons and pathways (Section 3.2). Thirdly, an overview of skeletal muscle modelling is provided, with a focus on contractile behaviour and the integration of sensory fibres (Section 3.3). Lastly, proprioceptive feedback modelling is described, closing the loop between the skeletal muscle and spinal circuit models (Section 3.4). This paper by no means aims to include a detailed description of all models, but to provide an overview of the modelling approaches taken in representing each component and how they can be connected.

### 3.1 Motor cortex models

The role of the motor cortex in the generation of movement, functionally and computationally, has not been resolved (Scott, 2008; Tanaka, 2016). Electrophysiological studies have provided insights into mapping the physiological structure of the motor cortex, but have not yet been able to provide a cohesive understanding of how the neural activity in the motor cortex is involved in producing muscle contraction. There have been two main theories about how neurons in the motor cortex relate to and produce movement. The previously held representational view sought to relate the firing of pyramidal neurons in the motor cortex to movement parameters such as muscle tension, direction and velocity of movement, joint angle, or EMG (Georgopoulos et al., 1986; Sergio and Kalaska, 1997; Reina et al., 2001; Cherian et al., 2011). Phenomenological approaches to modelling cortical activity in movement have therefore used kinematic variables, such as movement direction, as an input to describe the firing rates of upper motoneurons (Mussa-Ivaldi, 1988; Todorov, 2000). This approach is described in Section 3.1.2.

More recently, the dynamical systems perspective argues that individual neurons cannot code for movement parameters, but there may be patterns generated by the activity of populations of neurons which determine the motor output. For a summary on dynamical systems in modelling movements see Shenoy et al. (2013). Neuron activity has been modelled at different levels, from individual cells using biophysically-based models of neurons model action potentials (further described in section 3.1.1) to populations of neurons connected in networks (Wilson and Cowan, 1972; Brette and Gerstner, 2005; Jolivet et al., 2008; Abbott et al., 2016; Vyas et al., 2020). Recently, artificial neural network models have been used to model the complex dynamics found in cortical activity. Some of these are phenomenological in their representation of the motor cortex while others reconstruct detailed physiological structures (Hill and Tononi, 2004; Esser et al., 2005; Potjans and Diesmann, 2014; Michaels et al., 2020; Dura-Bernal et al., 2022). Neural network models are able to replicate the firing dynamics of the cortex during rest and movement. Firing rates of neurons in the cortex, even at rest, are highly irregular and asynchronous with long tailed distributions of interspike intervals (Churchland and Shenoy, 2007; Kumar et al., 2008; Tomov et al., 2014; Borges et al., 2020; Dabrowska et al., 2021) and these properties are thought to arise from, or be influenced by the properties of the neural network (Destexhe, 2011; Chen and Gong, 2019).

#### 3.1.1 Individual neurons

The initiation and propagation of action potentials in neurons were first quantitatively modelled by Hodgkin and Huxley in 1952 (Hodgkin and Huxley, 1952). Their model incorporated the sum of three currents (K^+^, Na^+^ and leak) to describe the membrane current (Eq. 1). The rapid, transient Na^+^ current is responsible for the ‘spike’ of the action potential and the K^+^ current is responsible for repolarising the neuron back to resting potential or a hyperpolarised, refractory state after an action potential. The proportion of ion-channels available as a proportion of the maximum conductance is described by three voltage and time dependent variables *m*, *n* and *h* in Eq. 1:

$$C_{\text{m}}\frac{dV}{dt}=I\left(t\right)-g_{\text{K}}\;n^{4}\;\left(V\left(t\right)-E_{\text{K}}\right)-g_{\text{Na}}\;m^{3}\;h\;\left(V\left(t\right)-E_{\text{Na}}\right)-g_{\text{L}}\;\left(V\left(t\right)-E_{\text{L}}\right).$$ (1)

Therein, *C*_m_ is the membrane capacitance, *V* is the membrane potential of the neuron, *I* is an applied membrane current, *g* is the conductance of the membrane (subscript denotes ion channel) and *E* is the equilibrium potential of the respective channels (denoted by subscripts).

In models with many neurons, Hodgkin-Huxley equations may be computationally expensive, and so simplified neuron models such as Izhikevich neurons, Fitz-Hugh Nagamo and leaky integrate-and-fire (LIF) neurons are used to reduce computational burden (Izhikevich, 2003; Yamazaki et al., 2022). These simplified neuron models neglect detailed ion-channel dynamics but are still able to describe the basic shape and timings of neuronal action potentials (Trappenberg, 2002). The leaky integrate-and-fire neuron takes the form:

$$\tau_{\text{m}}\frac{dV\left(t\right)}{dt}=-\left(V\left(t\right)-V_{\text{r}}\right)+\frac{I\left(t\right)}{C_{\text{m}}},$$ (2)

where *V* is the membrane potential, *V*_r_ is the value to which the membrane potential is reset after firing an action potential, *τ*_m_ is the time constant and *C*_m_ is the capacitance of the membrane. *I* represents the currents in and out of the cell due to post-synaptic potentials from other neurons or external microelectrodes (Dayan and Abbott, 2001; Gerstner et al., 2014) (Eq. 2).

#### 3.1.2 Representational approach

Motor cortex neurons have been found to be most ‘active’ for certain directions, exhibiting tuning curves of preferred directions (Georgopoulos et al., 1986). By representing the firing of individual neurons as vectors and then using a weighted vector sum, Georgopoulos et al. (1986) found that the resulting population vector was in a direction congruent with the direction of hand movement. The firing rate of neurons using their ‘preferred direction’ can be characterised by the following cosine tuning equation, where *fr*(*i*, *t*) is activity of neuron *i* at time *t*, *d*(*t*) is intended movement direction, *d*_pref_(*i*) is the preferred direction of the neuron, *fr*_0_ is the baseline firing rate and *g*_neuron_(*i*) is the gain of the neuron, i.e., a scaling factor representing the neuron’s sensitivity to input (Eq. 3):

$$fr\left(i,t\right)=fr_{\text{0}}+g_{\text{neuron}}\left(i\right)\;\cos\left[d\left(t\right)-d_{\text{pref}}\left(i\right)\right].$$ (3)

It has been suggested that the preferred direction of neurons is determined by the musculoskeletal system’s biomechanical properties (Hirashima and Nozaki, 2012; Lillicrap and Scott, 2013; Suminski et al., 2015). Neural activity in the motor cortex has been phenomenologically described in motor control models based on the assumption that neuronal firing rates are reflective of properties such as end effector position and velocity, joint torque or muscle-length (Mussa-Ivaldi, 1988; Todorov, 2000; Ajemian et al., 2008). Models by Mussa-Ivaldi (1988) and Todorov (2000) have demonstrated that neuron firing can also be described using functions of muscle length, shortening velocity, acceleration and force.

The general equation of phenomenological models of neuron firing rates is:

$$fr\left(i,t-\phi \left(i\right)\right)=f_{i}\left(p_{1}\left(t\right),p_{2}\left(t\right),\ldots ,p_{N}\left(t\right)\right),$$ (4)

where *fr*(*i*) is neuron activity, *ϕ*(*i*) is the neuron specific latency between cortex and muscle, *f*_*i*_ is a neuron specific input-output function, and *p*_*i*_ are movement parameters (Wang et al., 2022) (Eq. 4). These models have been able to reproduce the directional tuning curve of activity from recorded neuron populations but the correlations to kinematics and biomechanical properties may be epiphenomenal and do not capture the nuances in the wide range of individual neuron activity or represent the physiological connections of supraspinal circuits (Scott, 2000).

Modelling approaches using traditional recurrent neural networks (RNNs) to generate neural activity at the population level based on muscle activity, have also been implemented to capture both directional tuning properties and underlying rotational dynamics observed in neural firing data (Michaels et al., 2016; 2020; Sussillo et al., 2016). Rotational trajectories in neural firing data have been observed in the primate motor cortex during arm reaches following the application of a dimensionality reduction method, jPCA. When the first two principal components are plotted against each other, they show rotational trajectories which are thought to represent oscillatory or rhythmic patterns of activity in the brain. The criticisms of this approach is that rotational dynamics are not linked to physiological structure, mechanisms or behaviour and so lacks explanatory power (Lebedev et al., 2019). Traditional RNNs also use non-biophysically based models of ‘neurons’ and continuous values of activation rather than capturing the dynamics of physiological action potentials.

#### 3.1.3 Spiking neural networks

A neural network refers to a population of neurons as well as the synapses and connections between them. Spiking neural networks are artificial neural networks based on biologically-realistic models of neuron action potentials (described in the previous Section 3.1.1), which encode information in the coordinated timing of action potentials or ‘spikes’. Synaptic current models are used to represent the dynamics of the receptors or rate of ion flows in the pre- and/or post-synaptic neuron. Inhibitory synapses typically are stronger than excitatory synapses by two to six times (Alvarez and Destexhe, 2004; Xue et al., 2014; Gao et al., 2017).

The single exponential model of a post-synaptic current (*I*_syn_) acting on the membrane potential takes the general form of an exponential decay function with time constant *τ*_syn_ (Eq. 5):

$$\frac{dI_{\text{syn}}\left(t\right)}{dt}=-\frac{I_{\text{syn}}\left(t\right)}{\tau_{\text{syn}}}.$$ (5)

The connectivity, or topology, of neural networks and its influence on firing dynamics is a rich area of research (Larremore et al., 2011; Litwin-Kumar and Doiron, 2012; Bennett and White, 2021). Neurons in the brain exhibit local connectivity, but also have long range connections between areas. Small world or patchy network topologies where the distribution of connections are unevenly distributed or spatially defined may be more physiologically representative than a model with random connectivity (Wang and Chen, 2003; She et al., 2016; Card and Gharbawie, 2020). In the motor cortex there is broader intralayer connectivity with narrower, columnar interlayer connectivity and recurrent connections between layers, with a large number of connections from superficial layers to deep layers (Weiler et al., 2008; Hooks et al., 2013). The connectivity of a neural network model can have an architecture based on these connectivity principles, and experimental data from physiological experiments can also be used to inform and tune these connectivity parameters.

Potjans and Diesmann (2014) used previous experimental data to create a cortex model with complex connectivity within and between cortical layers which was able to capture the asynchronous, irregular spiking behaviour in larger, more realistic numbers of neurons and synapses (i.e., tens-of-thousands and millions respectively). Large-scale biologically-inspired spiking neural networks, containing populations of hundreds to tens-of-thousands of excitatory and inhibitory leaky integrate-and-fire neurons, have recently been used in models of motor cortical activity (Esser et al., 2005; Farokhniaee and Lowery, 2019; Rostami et al., 2020). These models have been able to replicate the spontaneous firing activity in the laminar structure of the cortex, the oscillatory rhythm in the condition of Parkinson’s disease and neural states involving movement (Farokhniaee and Lowery, 2019; Rostami et al., 2020). Spiking neural networks are more biologically realistic than traditional RNNs and so can provide a more mechanistic understanding about how cortical activity is generated. However, previous cortical models have not been linked with models of muscle contraction and feedback circuits in the spinal cord to represent the corticomuscular pathway.

#### 3.1.4 Corticospinal pathway

The descending activity from the motor cortex to neuron pools in the spinal cord has typically been modelled as direct inputs of firing rates or currents which represent the cumulative descending drive of the corticospinal tract and brain (Stienen et al., 2007; Sreenivasa et al., 2015; Mascaro et al., 2020; Volk et al., 2021). This is supported by experimental and simulation studies which suggest that the majority of the input to *α*-motoneurons are common to the pool (Negro et al., 2016b; Del Vecchio et al., 2022). However, recent work by Marshall et al. (2022), showed that motor units might be flexibly recruited according to task demands and suggested that the descending drive from the cortex may play a role in this ability. Previous models that have linked motor cortex or brain activity to spinal cord and muscle models have used the output of individual neurons or neuron groups to drive individual muscles (Teka et al., 2017; Mascaro et al., 2020; Pérez Fernández et al., 2021). Teka et al. (2017) used six neurons corresponding to each muscle in the upper limb arm model to directly control the spinal motoneurons. Pérez Fernández et al. (2021) used a more complex neural model of three layers representing the sensory input, interneurons and *α*-motoneurons, respectively, with 48 neurons in each layer. These models, however, do not capture the complex dynamics of firing in the motor cortex nor take into account any recurrent connections which are prevalent in the circuitry and subsequently play a role in *α*-motoneuron recruitment.

The previous subsections described approaches to modelling the upstream neural activity of the motor cortex. Here we provide an example of a motor cortex model using a spiking neural network that can provide the descending drive of the corticospinal tract and be integrated with downstream spinal cord and muscle models discussed in Section 2.3 and Section 3.4, paving the way towards modelling the connection from cortex to muscle. A more detailed description of this motor cortex model can be found in Haggie et al. (2022).

The cortex can be represented by a spiking neural network with populations arranged in a laminar structure. The connectivity within and between cortical layers is adapted from previous cortical models notably Esser et al. (2005) and Potjans and Diesmann (2014), which were based on experimental evidence. Individual neuron activity is described by a leaky integrate-and-fire (Eq. 2), to reduce computational expense allowing for more realistic numbers of neurons in networks, i.e., tens of thousands, and degrees of synaptic connections of each neuron, i.e., thousands, to be modelled. Parameters are described in Table 1.

**TABLE 1:** Table of example parameters for a spiking neural network model of motor cortex (taken from Potjans and Diesmann, 2014).

Symbol	Name	Value
Cortical Neurons		
*C*_m_	Membrane capacitance	250 pF
*θ*	Threshold value	−50 mV
*t*_ref_	Refractory time	2 ms
*τ*_m_	Membrane time constant	10 ms
*V*_r_	Reset value	−65 mV
Synapses		
*τ*_syn_	Synaptic time constant	10 ms
*w*_synE_	Weighted excitatory strength	1
*w*_synI_	Weighted inhibitory strength	−4
*S*_syn_	Synaptic strength	87.8 pA (SD 8.78 pA)
Delays		
Δ_e_	Excitatory delay	1.5 ms (SD 0.75 ms)
Δ_i_	Inhibitory delay	0.8 ms (SD 0.4 ms)

Subscripts denote excitatory (e) or inhibitory (i) neuron parameters. SD: standard deviation.

When the neuron membrane potential reaches threshold *θ*, the neuron fires an action potential. On the firing of an action potential in a pre-synaptic neuron, the post-synaptic neuron receives a change in synaptic current, $I_{\text{syn}}^{\text{post}}$ , of the synaptic strength value, *S*_syn_, scaled by a relative weighting value, *w*_syn_ (Eq. 6). The synaptic strength and the synaptic weight both contribute to the change in conductivity which influences the response of the post-synaptic membrane potential in response to the pre-synaptic action potential. The synaptic weight specifically represents the potential modulation of the post-synaptic transmission. This occurs after a delay Δ which accounts for the time it takes for the action potential to propagate down the axon. The value of $I_{\text{syn}}^{\text{post}}$ is described by a single exponential model in Eq. 5. After a neuron fires an action potential, its membrane potential is reset to *V*_r_. A neuron cannot fire an action potential to other neurons when it is within the time of the refractory period (*t*_ref_) since its previous action potential.

$$\begin{array}{ll} I_{\text{syn}}^{\text{post}}\left(t\right) & =I_{\text{syn}}^{\text{post}}\left(t\right)+S_{\text{syn}}\;w_{\text{syn}},\\ & \;\;\text{when pre‐synaptic neuron }V\left(t\right)\geq \theta . \end{array}$$ (6)

The firing frequency of a neuron is the number of times the neuron reaches the threshold over a timestep. The firing activity of a proportion of the layer V motor cortex neurons in this spiking neural network can then be used to represent the descending corticospinal tracts which synapse onto spinal interneurons and *α*-motoneurons in the spinal cord. Because the specific connectivity of the corticospinal tract to the spinal cord neurons is still unknown, this could be a probabilistic parameter based on spatial distances or tuned to experimental data of the input to *α*-motoneurons.

**Model inputs and outputs:** The model receives input from a Poisson distribution to maintain spontaneous activity during resting state, and an external stimulation or extrinsic currents can be applied to increase firing frequencies and produce the patterns of increased firing related to motor output depending on the research question. The output of this model would be the firing times of a subset of the group representing layer V excitatory pyramidal neurons which make up part of the axons in the corticospinal tract connecting the cortex to spinal circuitry.

### 3.2 Spinal circuitry models

In the spinal cord, descending motor commands, for example, from the motor cortex, as well as signals from peripheral sensory organs are integrated and processed by interneurons, Renshaw-cells and *α*-motoneurons, which subsequently activate muscle fibres (Section 2.2). To represent the spinal circuitry, populations of neurons and their connections within the spinal cord as well as to the periphery need to be defined according to the physiological pathways. Different modelling strategies for neurons are extensively discussed in Section 3.1 and so this section will focus on the specific neuron characteristics that need to be considered in the spinal cord.

#### 3.2.1 Spinal neurons

Models of neurons in spinal circuits mainly use two approaches to describe neuron behaviour. Transfer functions can be used to describe the input-output behaviour of neuron populations, following a phenomenological approach. For example, the relation between input, *α*_in_(*t*) ∈ [−1, 1], and the activity of a neuron population, *α*_out_(*t*) ∈ [0, 1], is typically described by a sigmoidal function (e.g., Raphael et al., 2010):

$$\alpha_{\text{out}}\left(t\right)={\left[1+\exp\left(-a\left[\alpha_{\text{in}}\left(t\right)-b\right]\right)\right]}^{-1},$$ (7)

where, *a* and *b* are parameters that are tuned to represent experimentally determined input-output behaviour (Eq. 7). Due to their low computational cost, transfer functions are well suited to investigate the interplay within large networks of neuron populations; however, transfer functions cannot adequately represent the spiking activity of single *α*-motoneurons (e.g., Raphael et al., 2010; Li S. et al., 2015; Teka et al., 2017; Parziale et al., 2020).

The second approach employs biophysical neuron models, which explicitly model the membrane dynamics of individual neurons in response to synaptic inputs (Section 3.1.1). Cisi and Kohn (2008) used the Hodgkin-Huxley formalism to describe *α*-motoneurons, interneurons and Renshaw-cells by a compartmental model considering the activity of a number of ion channels. Their model has been integrated in several models of spinal cord circuitry, e.g., by Dideriksen et al. (2015) and Elias et al. (2014). Variations of the standard integrate-and-fire model (Section 3.1.1) were employed by Sreenivasa et al. (2015), Stienen et al. (2007) and York et al. (2022), which is a common way to reduce the computational cost of modelling neuron populations. In the biophysical modelling approach, each neuron is modelled individually and governed by one set of equations. Neuron populations can be obtained by replicating the models according to the desired pool size. *α*-motoneurons that innervate the same muscle differ significantly from each other with respect to their properties and behaviour (Section 2.2). The model parameters for a pool of *α*-motoneurons can be obtained from an exponential distribution where the value of a parameter *p*_*i*_ of the *i*-th neuron of a pool of *N*_MN_ *α*-motoneurons is calculated from the equation:

$$p_{i}=p_{\text{low}}+\frac{p_{\text{up}}-p_{\text{low}}}{100}\;\exp\left(\ln\left(100\right)\frac{i}{N_{\text{MN}}}\right),$$ (8)

where *p*_low_ and *p*_up_ are the lower and upper extremes of the parameter’s value range, respectively (Fuglevand et al., 1993; Negro and Farina, 2011) (Eq. 8). *α*-motoneuron pools that follow the approach described above inherently account for physiological principles like the orderly recruitment following the size principle. Populations of interneurons and Renshaw-cells are usually described with a constant set of parameters (e.g., Cisi and Kohn, 2008; Sreenivasa et al., 2015). In Table 2 we provide an exemplary set of parameters for *α*-motoneurons and interneurons following the integrate-and-fire approach (Eq. 2).

**TABLE 2:** Example parameters for spinal cord neurons and synapses according to the standard leaky-integrate-and-fire modelling approach.

Symbol	Name	Value	Source
*α*-motoneurons			
*C*_m_	Membrane capacitance	81.14–375.62 pF	Sreenivasa et al. (2015)
*V*_r_	Reset potential	−70 mV	
*τ*_m_	Membrane time constant	7.83–13.36 ms	
Interneurons			
*C*_m_	Membrane capacitance	160 pF	Sreenivasa et al. (2015)
*V*_r_	Reset potential	−70 mV	
*τ*_m_	Membrane time constant	10 ms	
Synapses			
$\tau_{\text{syn}}^{\text{exc}}$	Excitatory synaptic time constant	0.5 ms	Finkel and Redman (1983)
$\tau_{\text{syn}}^{\text{inh}}$	Inhibitory synaptic time constant	1 ms	Stuart and Redman (1990)

#### 3.2.2 Spinal pathways

The neurons in the spinal cord receive inputs from different sources and are connected to each other via synapses, creating spinal pathways. Due to the large number of, partly still unknown, interactions between the neurons of the spinal cord, modellers must make a decision on which pathways shall be considered. Focusing on a single pathway such as the monosynaptic stretch reflex, which is based on the monosynaptic connection of muscle spindle Ia afferents and *α*-motoneurons, enables the investigation of pathways in isolation, but possible interactions are neglected (e.g., Schuurmans et al., 2009). Often, a system with two muscles, acting as agonist and antagonist, and their respective motoneuron pools and sensory organs are considered. Thereby, muscle spindles provide feedback to the neuron pools of both muscles, i.e., both the excitatory homonymous input to the agonist muscle and the reciprocal inhibition of the antagonist, enabling the coordination of behaviour based on sensory information (e.g., Sreenivasa et al., 2015). Considering further pathways, for example, the secondary afferents of muscle spindles or Ib afferents from Golgi tendon organs, can provide additional sources of sensory information. For those specific afferents, often only the connections to the homonymous muscle are considered (e.g., Elias et al., 2014; Dideriksen et al., 2015; Moraud et al., 2016; York et al., 2022). By implementing both mechanical and neural interactions between the simulated muscles, these approaches are suited to improving our understanding of strategies the central neural system could use to control the neuromuscular system. To investigate the spinal circuity in more detail, modellers could also consider the influences of Renshaw-cells, sensory pathways involving more than one interneuron (e.g., Stienen et al., 2007; Raphael et al., 2010; Buhrmann and Di Paolo, 2014; Parziale et al., 2020) and *γ*-motoneurons, which innervate muscle spindles (Li S. et al., 2015). These models can help researchers to understand how certain connectivity rules and their modulation enable the central nervous system to perform numerous different tasks with one anatomical kind of neuronal network.

In the spinal cord, information is exchanged between the cells via synapses. Physiologically, every spike of a pre-synaptic cell induces a local change of the membrane potential in the post-synaptic cell. The configuration of synapses in the model ultimately depends on the neuron model. Using the leaky integrate-and-fire approach, synapses can be defined as described in Section 3.1.4, Eqs 5, 6, where the time constant can be chosen according to experimental observations (Table 2). The synaptic strength in combination with the synaptic weight, previously defined in Section 3.1.4, defines the potency of the interaction, which can vary between pathways and movement tasks (Bawa and Sinkjaer, 1999; Baudry and Enoka, 2009; Yavuz et al., 2018). Thus, these parameters need to be adjusted to the applied scenario. The weights can be tuned empirically to obtain some desired motor output (Teka et al., 2017; Kapardi et al., 2022) or by employing an optimisation algorithm (Raphael et al., 2010). When considering populations of neurons, the connectivity rules can be based on the distance of the neurons to each other (e.g., Sreenivasa et al., 2015) or on stochastic distributions (e.g., Dideriksen et al., 2015).

Neural signals (action potentials) require a finite amount of time to travel from the sensory organs to their respective target neurons in the spinal cord. This means that sensory information is not provided to the neural system instantaneously. The delay in the transmission of action potentials is determined by the conduction velocity of the respective nerve and the anatomical distance between the muscle and the location of the neuron pool in the spinal cord. Conduction velocities for the nerve types named within this document can be found in Table 3.

**TABLE 3:** Conduction velocities for different types of nerves and their distribution within populations.

Nerve type	Conduction velocity	Distribution	Source
Corticospinal axons	5–94 m s^−1^	Exponential	Firmin et al. (2014)
Type I sensory	80–100 m s^−1^	Normal	Boyd and Kalu (1979), Heckman and Binder (1988)
Type II sensory	30–40 m s^−1^	Normal	Boyd and Kalu (1979)
Motor axon	70–110 m s^−1^	Exponential	Heckman and Binder (1988), Zengel et al. (1985)

**Model inputs and outputs:** The signals emerging from supraspinal centres as well as sensory organs serve as inputs to a spinal circuit model. For each arriving spike from pre-synaptic neurons, the target neurons receive a post-synaptic potential, i.e., current is injected into these neurons. The final output variables of this model component would be the firing rates $fr_{i}^{\text{MN}}, where\;\;i=1,\ldots ,N_{\text{MN}}$ , of the *α*-motoneurons, each innervating a specific set of muscle fibres.

### 3.3 Skeletal muscle models

In skeletal muscles, *α*-motoneuron axons synapse with muscle fibres at their neuromuscular junction and initiate excitation-contraction coupling. This leads, ultimately, to force generation that is transmitted to the skeleton via the connective tissues. There are a multitude of methods to model skeletal muscle. The following presents only a brief description of the modelling landscape, with a particular focus on muscle’s contractile behaviour. First, a general overview of muscle modelling is given followed by an exemplary model to demonstrate integration of sensory fibres.

Muscle models can be broadly classified as either phenomenological or biophysical, stemming from pioneering works of Hill (1938) and Huxley (1957), respectively. Phenomenological models are a superposition of lumped parameter functions that describe various muscle behaviours as black boxes at the macroscopic level and are typically based on Hill’s experimental observations (Section 3.3.2). Biophysical models consider the micromechanics and energetics of the interactions within the cross bridges and are typically based on Huxley’s sliding filament theory (Section 3.3.3). While the later models capture muscle behaviours such as the force-length in an emergent sense, i.e., based on micromechanical interactions, they are typically too cumbersome and computationally expensive to describe whole muscle behaviour. This has favoured the popularity of phenomenological models, especially in the simulation of limb or whole body movement.

For further reading on muscle modelling, see Dao and Tho (2018) or Röhrle et al. (2019) for 3D models (the latter including multiscale models), and Rockenfeller and Günther (2017b); Scovil and Ronsky (2006); Schmitt et al. (2019) for 1D models.

#### 3.3.1 Spatial modelling of skeletal muscles

Independent of the approach used to describe muscle behaviour, a muscle model usually requires a spatial component to predict movement, i.e., muscle forces must be directed and transmitted towards the skeleton to generate joint torques and limb movement. Muscles are typically modelled either as line-segments (1D models) or as volumes (3D models). One-dimensional models assume that force transmission within the muscle is solely characterized by the attachment points of the musculotendon complex. That is, a single force vector between attachment points describes whole muscle behaviour (e.g., Delp et al., 2007; Wu et al., 2016; Wochner et al., 2020). This idealisation of muscles provides a straightforward and computationally inexpensive method to model muscles, but comes at the cost of physiological accuracy.

On the other hand, 3D models treat the muscle as a volumetric solid and are able to capture varying fibre architecture and force transmission to the skeleton via attachment areas (rather than points) (e.g., Johansson et al., 2000; Blemker et al., 2005; Röhrle et al., 2017). While being more computationally expensive and often difficult to characterise, 3D models can reveal subtleties in muscle deformation not possible via 1D models, e.g., varying line-of-action, non-uniform muscle strains, contact with surrounding tissue, and surface deformations (e.g., Blemker et al., 2005; Wu et al., 2014; Röhrle et al., 2017; Weickenmeier et al., 2017; Ramasamy et al., 2018; Péan et al., 2019).

#### 3.3.2 Phenomenological approach: Hill-type models

Phenomenological models stem from the pioneering work of Hill (1938), who investigated the relationship between force production and contraction velocity. These models describe whole muscle behaviour and are typically composed of three elements: contractile element (CE), passive element (PE) and series elastic element (SEE), with the relationship (e.g., Zajac, 1989):

$$F_{\text{CE}}\left(t\right)+F_{\text{PE}}\left(t\right)=F_{\text{SEE}}\left(t\right).$$ (9)

where *F*_CE_(*t*) is the contractile element and represents the muscle active or contractile force, and *F*_PE_(*t*) and *F*_SEE_(*t*) are the parallel elastic and serial elastic elements and typically represent the connective tissues in the muscle and tendons, respectively (Eq. 9). Here, we focus on muscle’s active force and thus only consider the *F*_CE_ in the following (Eq. 10). The CE accounts for the hallmark behaviours of muscle contraction such as the force-velocity *f*_vel_(*t*) ∈ [0, 1] and force-length *f*_len_(*t*) ∈ [0, 1] relationships via superposition, (e.g., Gordon et al., 1966; Lloyd and Besier, 2003):

$$F_{\text{CE}}\left(t\right)=\alpha_{\text{musc}}\left(t\right)F_{\text{max}}\cos\left(\phi_{\text{penn}}\right)f_{\text{len}}\left(L\left(t\right)\right)f_{\text{vel}}\left(\dot{L}\left(t\right)\right).$$ (10)

Here, *L*(*t*) and $\dot{L}\left(t\right)=dL\left(t\right)/dt$ are muscle length and velocity, respectively, *F*_max_ is the maximum isometric force and *ϕ*_penn_ is the pennation angle. Lastly, *α*_musc_(*t*) ∈ [0, 1] is the muscle activity, which describes the average degree of muscle activation, with *α*_musc_(*t*) = 1 for full activation.

Eq. 10 assumes that the muscle properties, deformation, and activation are constant over the muscle. Therefore, such models lend themselves to a 1D representation of muscles, i.e., as line segments between attachment points. The low computational cost and simplicity of these models have led to their prevalence in the musculoskeletal biomechanical modelling community (e.g., Hatze, 1981; Zajac, 1989; Cheng et al., 2000; Scovil and Ronsky, 2006; Delp et al., 2007; Wu et al., 2016; Seth et al., 2018; Wochner et al., 2020).

Despite their prevalence, 1D muscle models are not without limitations. For example, it is not straight-forward to capture complex muscle architecture, model contact with surrounding tissues, or to consider the distributions of connective tissue and shearing within the muscle.

To overcome these limitations, the phenomenological approach has also been utilized with 3D geometries (e.g., Blemker et al., 2005; Wu et al., 2014; Röhrle et al., 2017; Weickenmeier et al., 2017; Péan et al., 2019). Here, local contractile stresses rather than total muscle force are considered. Then, Eq. 10 can be reformulated as (e.g., Johansson et al., 2000):

$$S_{\text{CE}}\left(t,X\right)=\alpha_{\text{musc}}\left(t\right)S_{\text{max}}f_{\text{len}}\left(\lambda_{\text{f}}\left(t,X\right)\right)f_{\text{vel}}\left(\dot{\lambda_{\text{f}}}\left(t,X\right)\right)M,$$ (11)

where *λ*_f_ = *L*(***X***)/*L*_0_(***X***) is the normalised fibre length or fibre stretch at position ***X***, and is the square of the invariant *I*_4_ of the Cauch-Green deformation tensor (Eq. 11). For the 1D case, the geometric line segment acts to direct *F*_CE_(*t*) towards the attachment points, whereas in the 3D case, muscle stress is directed along the local muscle fibre direction ***a*** (***X***) and is accounted for by the structural tensor ***M*** = ***a*** ⊗ ***a***. An exemplary force-length relationship (Röhrle et al., 2017) is given as;

$$f_{\text{len}}\left(\lambda_{\text{f}}\right)=\exp\left(-{\left|\frac{\frac{\lambda_{\text{f}}}{\lambda_{\text{f}}^{\text{opt}}}-1}{w_{i}}\right|}^{r_{i}}\right),$$ (12)

where $\lambda_{\text{f}}^{\text{opt}}$ is the optimal fibre length, *w*_*i*_ and *r*_*i*_ govern shape of the force-length relationship, where *i* = asc when $\lambda_{\text{f}}<\lambda_{\text{f}}^{\text{opt}}$ and *i* = dsc when $\lambda_{\text{f}}>\lambda_{\text{f}}^{\text{opt}}$ corresponding to the ascending and descending legs, respectively. Additionally, an exemplary force-velocity relation (van Leeuwen, 1991; Böl and Reese, 2008) is given as;

$$f_{\text{vel}}\left({\dot{\lambda }}_{\text{f}}\right)=\left\{\begin{array}{cc} \frac{1-{\dot{\bar{\lambda }}}_{\text{f}}}{1+q_{\text{c}}\;{\dot{\bar{\lambda }}}_{\text{f}}}\;\; & \text{if }{\dot{\lambda }}_{\text{f}}\leq 0,\\ d_{\text{v}}-\left(d_{\text{v}}-1\right)\;\frac{1+{\dot{\bar{\lambda }}}_{\text{f}}}{1-q_{\text{c}}\;q_{\text{e}}\;{\dot{\bar{\lambda }}}_{\text{f}}}\;\; & \text{if }{\dot{\lambda }}_{\text{f}}>0, \end{array}\right.$$ (13)

where ${\dot{\bar{\lambda }}}_{\text{f}}={\dot{\lambda }}_{\text{f}}/{\dot{\lambda }}_{\text{f}}^{\text{min}}$ is the stretch rate normalised to the minimum fibre stretch rate, and *q*_c_ and *q*_e_ are dimensionless characterisation parameters for the concentric and eccentric parts, respectively. Lastly, *d*_v_ is the degree of force enhancement during eccentric contractions. Exemplary material parameters for Eqs 12, 13 are given in Table 4.

**TABLE 4:** Material properties for the muscle contractile stress.

Symbol	Description	Value	Source
*S*_max_	Maximum isometric stress	0.09–0.21 N/mm^2^	Maughan et al. (1983), Kent-Braun and Ng (1999)
Force-length			
*λ*_opt_	Optimal sarcomere length	1.09 to 1.31	Saini and Röhrle (2022)
*w*_asc_	Shape param. (ascending branch)	0.32 to 0.36	
*r*_asc_	Shape param. (ascending branch)	2.4 to 3	
*w*_dsc_	Shape param. (descending branch)	0.23 to 0.37	
*r*_dsc_	Shape param. (descending branch)	1.4 to 2.2	
Force-velocity			
${\dot{\lambda }}_{\text{f}}^{\text{min}}$	maximum fibre stretch rate	−17 1/s	Böl and Reese (2008)
*q*_c_	Shape param. (concentric)	5	
*q*_e_	Shape param. (eccentric)	5	
*d*_v_	Eccentric force enhancement	1.5	

Typically, muscle activation (*α*_musc_(*t*); Eqs 10, 11) is treated at the whole muscle level, i.e., *α*_musc_ scales whole muscle activity—regardless of the dimensionality of the muscle model, i.e., 1D or 3D (e.g., Blemker et al., 2005; Delp et al., 2007; Péan et al., 2019; Wochner et al., 2020). A common approach to characterize *α*_musc_ is via a muscle’s electrophysiological signals. Here, electromyography (EMG) signals are rectified and smoothed and serve as a basis for muscle activity. However, since the EMG-to-force relationship is non-linear, a further mapping is used and is usually termed “activation dynamics,” i.e.,

$$\alpha_{\text{musc}}\left(t\right)=f\left(EMG\left(t\right)\right),$$ (14)

where *EMG*(*t*) is the rectified and smoothed EMG signal. While this is common place in 1D models (e.g., Hatze, 1981; Lloyd and Besier, 2003; Rockenfeller and Günther, 2017a; Walter et al., 2020), 3D models typically apply inverse dynamics to compute *α*_musc_(*t*) or impose it directly (e.g., Röhrle and Pullan, 2007; Péan et al., 2019).

While 3D models theoretically allow for spatial heterogeneity of muscle properties and activation, this is often overlooked. This means that the muscle volume is activated simultaneously (e.g., via Eq. 14), and no distinction is made between different fibre types. Recently, 3D models have included the spatial distribution of motor units by treating them in a volumetric sense, i.e., VF_*i*_(***X***) represents the volume fraction of motor unit *i* at position ***X*** in the muscle. Then, muscle activity may be computed by (e.g., Röhrle et al., 2019; Saini and Röhrle, 2023),

$$\alpha_{\text{musc}}\left(t,X\right)=\overset{N_{\text{MN}}}{\underset{i}{\sum }}\alpha_{i}\left(t\right)\;{\text{VF}}_{i}\left(X\right),$$ (15)

where *α*_*i*_(*t*) is the activity of motor unit *i*. Heterogeneous contractile properties can analogously be defined in relation to VF_*i*_(***X***). For example, the peak isometric stress (*S*_max_, Eq. 11), which is typically spatially constant, may be defined by

$$S_{\text{max}}\left(X\right)=\overset{N_{\text{MN}}}{\underset{i}{\sum }}S_{\text{max},i}\;{\text{VF}}_{i}\left(X\right),$$ (16)

where *S*_max,*i*_ is the peak isometric stress of motor unit *i*.

Note that Eq. 11 additively splits passive and active muscle behaviours and can be considered as a generalisation of the Hill-type model (Eq. 10). Alternatively, passive and active contributions can be multiplicatively split (at the level of the deformation gradient). For detailed discussion of the differences between the approaches, and a further development of hybrid approaches, see Klotz et al. (2021).

#### 3.3.3 Biophysical approach: Sliding filament models

Rather than taking a black box approach to muscle contraction, biophysical models simulate contractile mechanisms at the microscale. These models are primarily based on the sliding filament theory pioneered by A. F. Huxley (Huxley, 1957). Briefly, the starting point of the model is a fraction of attached cross bridges *n*_XB_ (*u*_XB_, *t*), where *u*_XB_ is the cross bridge displacement from the equilibrium (null force) position (along direction *x*). The conservation of cross bridges over an arbritary distance yields (e.g., Keener and Sneyd, 2009):

$$\frac{\partial n_{\text{XB}}}{\partial t}=\left(1-n_{\text{XB}}\right)\;f_{\text{atc}}\left(u_{\text{XB}}\right)-n_{\text{XB}}\;g_{\text{dtc}}\left(u_{\text{XB}}\right)+v\left(t\right)\;\frac{\partial n_{\text{XB}}}{\partial x}.$$ (17)

That is, the rate of change of cross bridges ${\dot{n}}_{\text{XB}}$ is the difference between attachment (1 − *n*_XB_) *f*_atc_(*u*_XB_) and detachment *n*_XB_ *g*_dtc_(*u*_XB_) plus the “inflow” of *n*_XB_ over *u*_XB_ with velocity *v* (where *v* > 0 is contraction). By assuming that a bound cross bridge acts as a linear spring with stiffness *κ*, total contractile muscle force can be computed by:

$$F_{\text{CE}}\left(t\right)=\alpha_{\text{musc}}\left(t\right)\;\rho_{\text{XB}}\;\kappa \int_{\infty }^{-\infty }u_{\text{XB}}\;n_{\text{XB}}\left(u_{\text{XB}},t\right)\;dx,$$ (18)

where *ρ*_XB_ is the total number of cross bridges with displacement *u*_XB_. Force production occurs via the molecular motor myosin, which interacts with actin and converts chemical energy into mechanical energy. Such models have been typically been used to study micromechanical contraction dynamics, as they become too cumbersome and computationally expensive at the whole muscle scale. Attempts have been made to simplify the partial differential equations in the Huxley type models to ordinary differential equations (e.g., Zahalak, 1981; Razumova et al., 1999). These models make certain *a priori* assumptions on the distribution of cross bridges *n*(*x*, *t*) and have been used at the whole muscle scale for 1D and 3D models (e.g., Gielen et al., 2000; Oomens et al., 2003; Hayashibe and Guiraud, 2013). In such models, muscle activity is typically a function of *α*-motoneuron firing times ( $fr_{i}^{\text{MN}}\left(t\right)$ , for *α*-motoneuron *i*) that control Ca^2+^ concentration, leading subsequently to cross bridge cycling (e.g., Razumova et al., 1999; Shorten et al., 2007). This can be broadly formulated as (c.f. Eq. 14)

$$\alpha_{\text{musc}}\left(t\right)=f\left(\;fr_{i}^{\text{MN}}\left(t\right)\;\right)\;i=1,\ldots ,N_{\text{MN}},$$ (19)

where *N*_MN_ is the total number of *α*-motoneurons. Note that the phenomenological approach can also be applied to compute muscle activity on the basis of individual motor unit activity (e.g., Fuglevand et al., 1993; Raikova et al., 2018).

The muscle models presented thus far do not consider action potential propagation along muscle fibres. This can be justified by assuming instantaneous propagation of the action potential, which has been shown to have a negligible effect on musculotendon force, when behaviour in the range of seconds is of interest (Schmid et al., 2019; Saini et al., 2022). However, action potential propagation must be modelled when sub-millisecond contractile behaviour is of interest or when electrophysiological modelling is required (e.g., Mordhorst et al., 2015; Klotz et al., 2022). The latter requires multi-physics modelling to compute potential fields at the macroscopic scale (e.g., Fernandez et al., 2005; Böl et al., 2011; Klotz et al., 2022) or within individual muscle fibres via multiscale modelling (e.g., Heidlauf and Röhrle, 2014; Mordhorst et al., 2015; Schmid et al., 2019). Multi-physics muscle models simulate action potential propagation via Hodgkin-Huxley type models (Hodgkin and Huxley, 1952). In multiscale models, muscle activity is a function of the local (half) sarcomere stress *γ*_sarc_, which itself is dependent on the percentage of cross bridges in the post-stroke state ${\bar{A}}_{2}\left(t\right)$ , and the rate of shortening ${\dot{\lambda }}_{\text{f}}$ along the fibre, parameterised by *s*, i.e., (c.f. Eq. 15),

$$\alpha_{\text{musc}}\left(t,X\right)=f\left(\;\gamma_{\text{sarc}}\left({\bar{A}}_{2}\left(t\right),{\dot{\lambda }}_{\text{f}}\left(s\right),X\right)\;\right).$$ (20)

#### 3.3.4 Integrating proprioceptive feedback

Mechanical quantities such as muscle stretch, rates of stretch, pressure, and tension throughout the musculotendon complex are sensed by proprioceptive sensory organs, which interact with the neural system, completing the feedback loop (Section 3.4). From a modelling perspective, these mechanical quantities are readily available from the muscle models.

Within the 3D framework, a distribution of muscle spindles can be defined within the muscle at locations $X_{k}^{\text{spindle}},\;\;k=1,\ldots ,N_{\text{spindle}}$ . Experimental data on the exact locations of muscle spindles is sparse; assuming a uniform distribution seems reasonable to ensure a good sensory coverage of the muscle. On the other hand, Golgi tendon organs (GTOs) are confined to regions adjacent to the myotendinous junctions and each GTO lies in series within a specific set of muscle fibres (Schoultz and Swett, 1972), i.e., at locations $X_{j}^{\text{GTO}},\;\;j=1,\ldots ,N_{\text{GTO}}$ confined to the myotendinous region. These locations are associated with a set of fibres for 3D multiscale models and with a given volume (with fibre orientation) for 3D macroscopic models. The mechanical variable of interest can be extracted in a straightforward manner at these locations at any given time, e.g., stretch and rate of stretch may be used for the muscle spindles, i.e., $\lambda_{\text{f}}\left(X_{k}^{\text{spindle}},t\right),\;{\dot{\lambda }}_{\text{f}}\left(X_{k}^{\text{spindle}},t\right)$ , or fibre tensile stress for Golgi tendon organs, i.e., $S\left(X_{j}^{\text{GTO}},t\right)$ . Note that *λ*_f_ does not require the modelling of individual fibres (i.e., multiscale model), and can be approximated by local deformation in continuum mechanical models, or by whole muscle deformation in 1D models (see below). Furthermore, since many Golgi tendon organ models take total muscle force as input (Section 3.4.2), the local stresses need to be converted to a singular force value that represents total muscle force. This may be achieved by defining a surface over the myotendinous junctions and calculating the total force from the local stresses and the plane’s cross-sectional area, i.e., *F*_musc_ = *∫*_*S*_ ***S*** (***X*** ^GTO^)*dS*.

In 1D models, the entire muscle experiences the same deformation and thus only an averaged sense of muscle stretch can be obtained, i.e., *λ*_f_(*t*) = *f*(*L*(*t*)/*L*_0_(*t*)), where *L*_0_ and *L* are the resting and current muscle lengths, respectively. That is, all muscle spindles experience the same deformation. Computing GTO activity is similarly straightforward given that force at the myotendinous junction must equal total muscle force (at static equilibrium) since the musculotendon-complex is modelled as a 1D force vector.

**Model inputs and outputs:** Typical inputs to muscle models are spike trains of the *α*-motoneuron pool. Typical outputs of the muscle models are contractile forces and length changes (for non-isometric contractions). Muscle models may either be biophysical or phenomenological or a combination of the two (i.e., multiscale models), and either 1D or 3D.

### 3.4 Proprioceptive feedback models

The preceding sections dealt with efferent activity in the descending corticomuscular pathway, through which signals from the motor cortex are propagated through the spinal circuitry and culminate in muscle contraction. The afferent or ascending pathway proceeds in the opposite direction, where proprioceptive information from sensory organs embedded in the musculotendon complex are carried “upstream” towards the spinal cord and motor cortex. Muscle spindles and Golgi tendon organs are the major sources of proprioceptive feedback directly modulating muscle activity. Thereby, muscle spindle activity excites and GTO activity inhibits the *α*-motoneurons of the parent muscle (Section 2.4). This section will describe the contributions of these two types of sensory organs to the corticomuscular pathway and the most frequently used modelling approaches.

#### 3.4.1 Muscle spindles

Muscle spindles are specialised organs that sense muscle stretch and stretch velocity. There exists a range of phenomenological models that provide mathematical functions to describe the experimentally derived relationship between muscle stretch and the resulting change in afferent firing rate (Prochazka and Gorassini (1998) and references therein). Prochazka and Gorassini (1998) provides a generalised model for the muscle spindle Ia firing rate *fr*_Ia_(*t*):

$$fr_{\text{Ia}}\left(t\right)=4.3\;v_{\text{fib}}{\left(t\right)}^{0.6}+u_{\text{fib}}\left(t\right)+{\bar{fr}}_{\text{Ia}},$$ (21)

where the firing rate depends on the muscle fibre displacement *u*_fib_(*t*), muscle fibre velocity *v*_fib_(*t*) and the mean firing rate ${\bar{fr}}_{\text{Ia}}$ . Fibre deformation information may be derived via a muscle biomechanical model (i.e., Section 3.3.4). This model has been able to replicate experimentally recorded muscle spindle firing rates for a range of stretch velocities, however, it ignores the modulation of spindle sensitivity by the fusimotor system.

Maltenfort and Burke (2003) and Schmid et al. (2022) proposed another phenomenological model, which calculates the Ia afferent firing rate not only in response to muscle stretch, but also in response to fusimotor activation. Therefore, separate Ia firing rates in response to passive stretch, and static and dynamic fusimotor input are calculated, then the contributions in response to fusimotor drive are summed such that the higher rate partially suppresses the lower rate before being added to the passive contribution.

Mileusnic et al. (2006) and Lin and Crago (2002b) both created so called semi-physiological models, by explicitly modelling the anatomical structure of the spindle, i.e., the three intrafusal fibre types and their contributions to Ia and II afferent firing rates as well as their sensitivity to fusimotor input. In the physiological spindle, the contributions of each of the intrafusal fibres to the overall firing rate are summed non-linearly, meaning that the lower firing rates are partially suppressed by the higher rates, which is known as occlusion (Schäfer, 1974; Banks, 1994). The model by Mileusnic et al. (2006) considers this appropriately, while in comparison the larger rate in the model by Lin and Crago (2002b) completely suppresses the lower rate. Recently, Blum et al. (2020) published a sophisticated muscle spindle model, based on the contraction mechanism of the intrafusal fibres and their interaction with the muscle-tendon complex. The model is able to predict many experimentally observed activation- and history-dependent patterns of Ia afferent firing without explicitly modelling them. However, due to its complexity, it is expected to exceed the requirements for a muscle spindle model in a framework focusing mainly on the corticomuscular pathway from cortex to contraction.

Commonly, muscle spindle models represent the afferent firing rates as continuous frequency values. However, for the integration in spike-based spinal circuitry models, the signals have to be converted into spike trains. A Poisson process such as that described by Vannucci et al. (2017) or alternatively a renewal process (Gerstner et al., 2014; Ross, 2014) can be used. This means that the variability of the inter-spike intervals of individual spindles can also be considered (e.g., Burke et al., 1979; Nordh et al., 1983). It must be noted that the described muscle spindle models are parameterised with experimental data of muscle spindle firing rates recorded from cats. To represent human muscle spindle firing, which is slower than in cats, the frequency can be scaled (e.g., Dimitriou and Edin, 2008; Grandjean and Maier, 2014).

**Model inputs and outputs:** The choice of the muscle spindle model is coupled to the types of pathways that shall be considered within the spinal circuitry model. Depending on the model, muscle fibre stretch, its first and second time derivatives as well as activity of static and dynamic *γ*-motoneurons modulate the firing rates of the muscle spindle’s Ia and II afferents. It might be necessary to convert the firing rates into spike trains to pass the information to respective target neurons in the spinal cord.

#### 3.4.2 Golgi tendon organs

Golgi tendon organs (GTO) are located at the muscle-tendon interface and sense the force produced by the muscle fibres to which they are connected (Section 2.4). To our knowledge the only biophysically representative GTO model that relates single GTO activity to the contraction of specific muscle fibres was presented by Mileusnic and Loeb (2006). The integration of this model in the corticomuscular pathway, requires the muscle model to provide the tension in single muscle fibres and their assignment to specific motor units.

According to Al-Falahe et al. (1990), the ensemble firing rate of all GTOs in a muscle is a better representation of muscle force than the activity of single GTOs. Thus, several so called ensemble or population models have been proposed (e.g., Lin and Crago, 2002a; Mileusnic and Loeb, 2009; Crago, 2018) that relate total muscle force to the summed, or alternatively averaged, Ib afferent firing rate. The model by Lin and Crago (2002a), which is based on a three step filtering process, is the only one which considers the effect of muscle tension history. However, if this is not relevant for the intended modelling approach, a computationally more efficient approach such as a polynomial function can be used. A saturating function (Eq. 22) as proposed by Mileusnic and Loeb (2009) and Crago (2018) is able to predict the relation between total muscle force *F*_musc_(*t*) and (total or mean) Ib afferent firing rate *fr*_Ib_(*t*), wherein, *F*_max_ denotes the maximum voluntary muscle force:

$$fr_{\text{Ib}}\left(t\right)=a\;{\left(\frac{F_{\text{musc}}\left(t\right)}{F_{\text{max}}}\right)}^{b}+c.$$ (22)

The parameter *b* determines the steepness of the function and takes on values between 0.2 and 0.26 (Mileusnic and Loeb, 2009; Crago, 2018), and the parameters *a* and *c* determine the lower threshold for sustained firing and the maximum rate at maximum muscle force. Muscle force may be derived via a muscle biomechanical model (i.e., Section 3.3.4). These parameters can be tuned to correspond to the range of frequencies recorded in human subjects (e.g., Dimitriou and Edin, 2008).

Note that, to convert the Ib afferent frequency values into a spike train, a Poisson or renewal process can be employed, similar to the muscle spindle models (Section 3.4.1).

**Model inputs and outputs:** The Golgi tendon organ model calculates the firing times of Ib afferents with reference to the total (or alternatively local) muscle force. Similar to muscle spindle afferents, it might be necessary to convert the firing rates into spike trains to pass the information to the respective target neurons in the spinal cord.

## 4 Discussion

In the preceding sections, we presented the modelling landscape of the corticomuscular motor pathway: the motor cortex, the spinal cord circuits, skeletal muscles and proprioceptive feedback systems, with a particular focus on their integration. System level models are important, particularly in the fields of motor control and biomechanics, because studying complex physiological behaviour such as movement involves many elaborate, complex, and hierarchical interactions and mechanisms. Individual components have usually been modelled in isolation by phenomenological and biophysical descriptions. However, there are very few studies which have taken a holistic approach to corticomuscular pathway modelling, i.e., between cortex and contraction.

Existing models of the corticomuscular pathway, which have considered the motor cortex and spinal network in conjunction with biomechanical models of muscle have typically used individual neurons or motor units to control each muscle separately. Simplified circuits of the spinal cord and connectivity from the cortex are used, which is understandable given that these connections are intricate and challenging to define. However, building on state-of-the art models of each component along the corticomuscular pathway and integrating them will result in a more realistic model to explore motor control.

Simplifying components of the corticomuscular pathway such as descending drive or motor unit recruitment can result in specific nuanced behaviour being missed. For example, the use of single *α*-motoneurons and motoneuron pools to represent an entire muscle means that questions regarding how recruitment might change within the MN pool, which occurs in fatigue, ageing and disease states, cannot be answered. Increasing the complexity of previous models, such as that by Teka et al. (2017), which used simplified single neuron models to represent descending drive and simplified motor unit pools to model contraction of individual Hill-type muscle models in the arm with an integrated spinal network, means that more complex behaviours and strategies used by the human motor control system can be explored. A key suggestion from the work by Teka et al. (2017) was that the afferent feedback from spinal networks played a significant role in the directional firing of motor cortex neurons, which highlights the potential interaction of subsystems in emergent phenomena. However, ignoring the behaviour of populations or networks of neurons by only having simplified, single neuron representations makes it difficult to address questions about the role of motor cortex.

A more realistic model of the corticomuscular pathway is needed to provide a mechanistic understanding of the entire system. Recent studies have discussed “closing the loop” between the computations of the brain and biomechanical representations of body (James et al., 2018; Mascaro et al., 2020; Pérez Fernández et al., 2021). James et al. (2018) integrated detailed brain circuits and biomechanical models in saccadic eye movements and Pérez Fernández et al. (2021) addressed the vestibular-ocular reflex. However, extraocular motor units use unique recruitment and contractile characteristics and are hard to compare to generalised limb movements (Shall and Goldberg, 1992). A more generalisable model which incorporates typical corticospinal and proprioceptive circuits of limb muscles would be more useful in applying the model to understanding neuromuscular diseases involving the corticomuscular pathway.

Mascaro et al. (2020) described a methodological framework in which combining experimental data with computational models can be used to construct and update model parameters, as well as drive experimental design. The framework by Mascaro et al. (2020) used experimental data from calcium imaging and electrophysiological recordings to drive and tune a mouse model which combined a spinal cord and biomechanical model of the upper limb, but did not yet include the motor cortex. Including the motor cortex would be a step further in modelling the entire corticomuscular pathway. In a similar framework with a human model, experimental data for model tuning and testing could be derived from non-invasive means such as TMS, functional near-infrared spectroscopy (fNIRS), electroencephalography (EEG), and EMG, as well as available invasive electrophysiological recordings such as that taken from micro-electrocorticography (*μ*ECoG) of epilepsy patients. The characterisation and validation of an integrated corticomuscular pathway model poses a major challenge, this will be further discussed in Section 4.2.

The work outlined here lays out the beginning of a framework for integrating the motor areas upstream from the spinal cord in biomechanical models and reinforces the constructive synergy of experimental and modelling research. An important consideration to build and run these models is a suitable implementation environment. An overview of existing platforms is outlined in Section 4.1. The integration of complex, physiologically based models of the motor cortex, spinal cord, muscle and sensory feedback organs can build understanding about the corticomuscular pathway at the biophysical level with the continued use of experimental data and testing (Section 4.2).

### 4.1 Implementation

There are a plethora of model implementations, software packages, and programs for each of the corticomuscular pathway components, available both in the literature and commercially. The selection of a particular modelling tool is challenging and is often guided by a mixture of practical (e.g., cost and availability) and research considerations. The wide range of available options hinders collaboration within the modelling community as modelling tools typically remain isolated, leading to model duplication and impeding validation and verification. Compounding this issue is a poor uptake of strong open-source policies by journals and research groups regarding the availability of the modelling outputs. Therefore, most models are implemented and kept in-house, using general purpose programming languages such as Fortran, C++, Matlab, or Python.

Despite this, certain software tools have gained prominence within the neuromuscular modelling community. For example, modelling the brain with neural network packages such as NEURON (Hines and Carnevale, 2001), NEST (Diesmann and Gewaltig, 2001), BRIAN2 (Stimberg et al., 2019), or MIIND (Osborne et al., 2021); or modelling musculoskeletal mechanics with OpenSim (Delp et al., 2007), or AnyBody Modelling System (AnyBody, Aalborg, Denmark) for 1D muscle models; or FEBio (Maas et al., 2012) or OpenCMISS (Bradley et al., 2011) for 3D models. An overview of software tools is provided in Table 5. In addition to these tools, databases provide important steps towards model unification and data sharing at the level of the individual motor pathway components. For example, the Musculoskeletal Atlas Project (MAP) (Zhang et al., 2014) aims to standardise musculoskeletal geometries and ModelDB provides a model exchange platform for computational neuroscience (McDougal et al., 2017). Also, The Allen Institue (Brain Map and Neurodata Without Borders) (Oh et al., 2014) and eBrains (Human Brain Project) (Schirner et al., 2022) provide a wide range of data and tools for brain-based research and technology development. BioModels hosts a broad collection of mathematical models of biological systems (Malik-Sheriff et al., 2020).

**TABLE 5:** A list of selected software tool for the implementation of the corticomuscular motor pathway.

Name	Neurons		Muscle		Sensory organs	Source
	Single cells	Neural networks	1D	3D		
NEST	*✓*	*✓*				Diesmann and Gewaltig (2001)
OpenCOR	*✓*					Garny and Hunter (2015)
BRIAN2	*✓*	*✓*				Stimberg et al. (2019)
NEURON	*✓*	*✓*				Hines and Carnevale (2001)
MIIND		*✓*				Osborne et al. (2021)
OpenSim			*✓*			Delp et al. (2007)
Anybody*			*✓*			AnyBody Technology, Denmark
LS-DYNA*			*✓*			Ansys Inc., United States
FEBio				*✓*		Maas et al. (2012)
OpenCMISS	*✓*			*✓*		Bradley et al. (2011)
ReMoto	*✓*	*✓*			*✓*	Cisi and Kohn (2008)
OpenDiHu	*✓*			*✓*	*✓*	Maier et al. (2019)
NEUROiD	*✓*	*✓*	*✓*		*✓*	Iyengar et al. (2019)

*✓* indicates that the tool is suited, by default, for the implementation of the respective pathway component. Commercial software tools are marked with an asterisk (*).

Although most modelling tools can interface with others via application programming interfaces (APIs), this often comes with modelling limitations, or involves added model configuration effort and computational costs. Here, standardisation of models and their inputs and outputs is key to unifying motor pathway models and fostering exchange and communication within the modelling community. For example, the Human Physiome project (Nickerson et al., 2016) seeks to standardise computer models across physiological systems (including the neural, spinal and musculoskeletal systems) and across scales. The Physiome Model Repository (PMR) (Yu et al., 2011) provides a platform to supply standardised modelling components for the motor pathway. Beyond that, the Physiome journal offers a means to publish mathematical models of physiologial components in a peer-reviewed journal, thereby ensuring model availability and reproducibility. Additionally, projects such as SimTK (National Institutes of Health, United States) provide a platform to share software, data, and models for the biomedical modelling community. Recently developed modelling tools enable the modelling of the integrated motor pathway in a single environment (e.g., Iyengar et al., 2019; Maier et al., 2019). While these tools currently exclude cortex modelling, they take steps towards a fully integrated motor pathway modelling environment.

In neuroscience, there have been recent advances in capturing and processing large sets of neural data recorded from microelectrode arrays, even in real time (Franke et al., 2012; Robinson et al., 2013). Newman et al. (2012) developed the NeuroRighter API which can be used to carry out ‘closed-loop’ experiments in which dynamic, real-world behaviour can be linked to electrophysiological measurements of brain activity. Taking a “Model-in-the-loop” approach (Potter et al., 2014) to combine model building with experiments is necessary for hypothesis testing and generation, data collection, and model validation.

### 4.2 Model validation

It is important to validate physiological models by comparing output variables or behaviours to experimental data. This poses a challenge in validating corticomuscular pathway modelling methods since the neuromuscular system remains relatively inaccessible to non-invasive methods. However, this lack of experimental data provides an opportunity to use modelling to inform and guide experimental studies and to increase our understanding of the corticomuscular pathway in an iterative fashion, i.e., formulating theories, building models, and conducting experiments, the findings of which can refine theories.

Motor pathway components have largely been validated in isolation. At the system or corticomuscular pathway level, however, what remains a challenge is the validation of the interactions and overall behaviour. Coupling previously validated models of individual components means that new parameters of connectivity will arise. This can result in new emergent behaviours and interactions, resulting in behaviours or outputs which need to be systematically validated. The modelling frameworks that combine spinal circuits, muscles and sensory organs already provide various coupling strategies and can be referred to for respective validation data (e.g., Stienen et al., 2007; Raphael et al., 2010; Dideriksen et al., 2015; Sreenivasa et al., 2015; Moraud et al., 2016; Kapardi et al., 2022).

This leaves two challenges for the validation of a model of the corticomuscular pathway. First, experimental studies which guide the coupling of the motor cortex and the spinal circuits have to be identified. Second, the overall system behaviour, ideally involving the action of all model components, has to be validated by comparing the simulated motor output to those of appropriate experimental studies.

There are many techniques used in the field of connectomics such as anterograde and viral tracers which are used to map the connections of neurons (Rathelot and Strick, 2009; Saleeba et al., 2019). However, only in simpler organisms such as a roundworm, *C. Elegans* (Varshney et al., 2011), has the full connectivity of neurons been mapped; the mouse brain has also been a recent focus of large-scale research projects (Oh et al., 2014; Erö et al., 2018). The sheer scale and complexity of mapping the human nervous system proves to be a challenge, though methods in electron microscopy and immunohistochemistry can be useful in determining synaptic pathways in the cortex and spinal cord (Saleeba et al., 2019 for a review on these methods). Invasive techniques for measuring data from neuron activity such as electrode arrays and calcium imaging (Trautmann et al., 2021) can be used, as well as intramuscular and intracellular electrodes (e.g., Powers and Binder, 1985; Zaback et al., 2022). Perturbations through pharmacological blockades, optogenetics, or localised lesions in animal models can also provide experimental evidence to test disruptions and resulting behaviour in the corticomuscular pathway model (Ziemann, 2013; Watanabe et al., 2020).

Non-invasive brain stimulation techniques such as transcranical direct current stimulation (tDCS), TMS and transcranial alternating current stimulation (tACS) can be used to stimulate activity in the motor cortex and investigate the downstream effects. For example, different protocols of strength and timings of TMS experiments can have facilitatory or inhibitory effects on the size of the motor evoked potentials (MEP) recorded by electromyography (EMG) (Ni et al., 2011; Volz et al., 2015). This can be combined with other experimental manipulations of excitation or inhibition, for example, through pharmacological blockades, to develop and test theories about circuits in the corticomuscular pathway (Ziemann, 2013). In addition, peripheral nerves can also be strategically stimulated with direct current stimulation to modulate spinal circuitry (e.g., Pierrot-Deseilligny, 2002) and high-density EMG can be utilised to analyse the activity of motor units (e.g., Yavuz et al., 2018). These experimental methods can be used in the model development loop to validate and fine tune the parameters in building the corticomuscular pathway circuitry.

### 4.3 Opportunities

Integrating existing knowledge about the motor system in a single system model means that we can potentially explain many phenomena involving different types of human movements and movement pathologies. A model encompassing the corticomuscular motor pathway will be able to contribute to explaining a wide range of electrophysiological findings related to health and disease, such as the task-dependent alteration of the motor unit recruitment order (e.g., Desnedt and Gidaux, 1981; Formento et al., 2021). Furthermore, it can also be of great benefit to provide a model of neurosurgical procedures, for example, selective dorsal rhizotomy for the reduction of spasticity in cerebral palsy patients by selectively cutting dorsal afferent fibres carrying sensory feedback to the spinal cord, or for the investigation of other “upper” or “lower” motoneuron diseases to compare and understand the causes of spasticity and flaccidity. Models may also help with the interpretation of responses to brain stimulation such as the effect of TMS protocols on motor evoked potentials as well as shed light onto the significance of direct, corticomotoneuronal connections on motor control, a characteristic unique to humans as well as higher primates (Lemon, 2008). An integrated modelling framework can also be used to investigate the effect of traumatic events on the corticomuscular pathway such as spinal cord injury or ischemic stroke. Continued development and improvement of the modelling frameworks, for example, by extending sensory feedback loops to the cortex, can also enable the investigation of the contribution of the cortex to long-latency responses of reflexes (Matthews, 1991; Reschechtko and Pruszynski, 2020).

A model of the corticomuscular motor pathway combines the fields of computational neuroscience and biomechanics. To continue the development of integrated models in motor control, experts from experimental neuroscience, connectomics, computer science, electrical engineering and mathematics will need to collaborate, providing an opportunity to open up new fields of research. Including clinicians in the model development process is an opportunity to guide clinically relevant questions, experiments and applications and can promote use in relevant cases. Models such as the one proposed in this text, which combine multiple subsystems from the brain to the muscle, can direct experiments, allow us to test theories and thereby develop new concepts and hypotheses of human motor control.

## 5 Conclusion

The motor pathway is composed of multiple interrelated and intricately connected subsystems. Therefore, knowledge of individual components cannot illuminate properties of the entire system since the interrelations themselves are responsible for emergent behaviours. So far, the field of computational biomechanics has tended to focus on motor output, i.e., the properties of muscle and the peripheral nervous system, while the field of computational neuroscience has tended towards the central nervous system, brain regions, and the properties of neural networks.

Modelling the corticomuscular motor pathway from motor cortex to muscle contraction is the next step in integrating neuroscience models of neurons and brain activity with biomechanical models of muscles in the body. This allows microscale models of cells to be expanded upon to allow for the exploration of larger meso- and macroscale systems. For each component in the corticomuscular motor pathway, the motor cortex, the spinal cord, the muscle, and its proprioceptors, a variety of modelling approaches already exists. Choosing the type and complexity of the model and components will depend on the scientific questions asked or the application in which it is to be used.

A major challenge which remains in modelling the corticomuscular pathway is being able to characterise the connectivity between the parts and so the gathering of additional experimental evidence will be vital to provide plausible parameters and validation. This endeavour requires a team of interdisciplinary experts to direct and test new theories. Creating integrated models means that how each of these different components interact and contribute to complex behaviours can be explored which will develop our understanding of motor pathologies and the human motor system.

## Data Availability

The original contributions presented in the study are included in the article/supplementary material, further inquiries can be directed to the corresponding author.
